# Quantitative Assessment of SBS-Modifier Content in Bituminous Binders Using Infrared Spectroscopy

**DOI:** 10.3390/polym18080898

**Published:** 2026-04-08

**Authors:** Saltanat Ashimova, Yerik Amirbayev, Adiya Zhumagulova, Manarbek Zhumamuratov, Sakypzhamal Begaliyeva, Zhanar Baibolekova, Mariya Smagulova

**Affiliations:** 1 Department of Science and Innovation Development, “Kazakhstan Road Research Institute” JSC, Astana 010000, Kazakhstan; s.ashimova@qazjolgzi.kz (S.A.); e.amirbaev@qazjolgzi.kz (Y.A.); s.begalieva@qazjolgzi.kz (S.B.); z.baibolekova@qazjolgzi.kz (Z.B.); m.smagulova@qazjolgzi.kz (M.S.); 2 Department of Technology of Industrial and Civil Engineering, L.N. Gumilyov Eurasian National University, Astana 010008, Kazakhstan

**Keywords:** polymer-modified bitumen, SBS modifier, infrared spectroscopy, FTIR-ATR, calibration curve, bituminous binders, quality control

## Abstract

Polymer-modified bituminous binders are widely used in road construction due to their enhanced mechanical performance; however, the effectiveness of these materials critically depends on the actual concentration of polymer modifiers, particularly styrene-butadiene-styrene (SBS). This study aims to develop and validate a rapid, reproducible Fourier Transform Infrared Spectroscopy—Attenuated Total Reflectance (FTIR-ATR) spectroscopy method for the quantitative determination of SBS content in polymer-modified bitumen (PMB). Since, to date, there is no clearly defined method for controlling the quantitative content of polymers in PMB, this creates difficulties in accepting the roadway into operation. Calibration PMB samples containing 1–4% SBS were prepared, tested for physical and mechanical properties, and analyzed spectroscopically to identify characteristic absorption bands at 966 cm^−1^ and 699–760 cm^−1^. A first-order calibration model was constructed to relate peak intensity to polymer concentration. The results demonstrate a clear linear correlation between SBS content and IR absorption features, confirming the suitability of FTIR as an instrumental method for routine laboratory control. Application of the model allowed determination of actual polymer mass fraction with high accuracy and reproducibility. The findings also showed that increased SBS levels improve softening point, elasticity, and low-temperature resistance, with 3–4% representing a performance-optimal range. Overall, the proposed FTIR-based approach provides an objective and efficient tool for quality control of polymer-modified binders and supports broader standardization efforts in the field.

## 1. Introduction

Bitumen plays a central role in road construction and maintenance in Kazakhstan, serving as the primary binding component in asphalt mixtures. From the standpoint of construction materials science, road bitumen is a complex organic substance derived from petroleum refining [1,2]. It consists of high-molecular-weight hydrocarbons and their derivatives, including asphaltenes, resins, and oils. These constituents govern its viscosity, adhesion, and resistance to temperature fluctuations, ultimately determining the durability and performance of pavement structures—an especially important factor given Kazakhstan’s diverse climatic conditions [3,4].

The article [5] describes practical applications of IR spectroscopy for bitumen testing, which include the evaluation of several types of binders prepared under controlled conditions, i.e., with a known polymer concentration and degree of aging. For comparison purposes, we also investigated some commercial bitumen available on the Polish market and those currently used for the construction of road surfaces.

Understanding the physicochemical properties of bitumen enables the optimization of its use in road engineering, ensuring long-term reliability and service life of asphalt pavements. This topic has been widely explored in both domestic and international research [6,7]. Polymer-modified bituminous binders (PMBs), produced through the incorporation of thermoplastic elastomers such as SBS and Styrene-Butadiene Rubber (SBR), ethylene-vinyl acetate (EVA), or polyolefins, significantly enhance pavement performance—improving fatigue resistance, rutting resistance, elasticity, and thermal stability. However, the operational characteristics of PMBs are highly sensitive to the actual polymer content [8]. Insufficient modifier dosage leads to degradation of performance, whereas excessive polymer content may cause phase instability and segregation.

Determining the actual polymer concentration in PMBs is therefore a critical step in quality control during production, acceptance procedures, and long-term pavement performance assessment. Despite the widespread use of polymer-modified binders, no universally accepted standardized method currently exists for the quantitative determination of SBS, SBR, EVA, or amorphous polypropylene polymers (APP) in bitumen [9,10].

Several approaches have been proposed to quantify polymer content in bitumen, yet their accuracy and reproducibility vary considerably. Contemporary scientific literature generally distinguishes four main methodological groups [11]:

1. Methods based on functional performance characteristics (rheology);
2. Dissolution and phase-separation techniques;
3. Chromatographic methods (GPC);
4. Infrared spectroscopy, which is considered the most promising direction.

The polymer concentration in PMBs governs the formation of a three-dimensional elastic network, resistance to aging, high-temperature rheological behavior, low-temperature elasticity, and overall durability under mechanical loads.

The article [12] discusses the issue of determining the SBS content in PMB, where the effect of the SBS copolymer on the physical and rheological properties of bitumen has been thoroughly studied and widely described. Based on laboratory tests, it was found that spectroscopy in the mid-infrared region is the most effective analytical method. With its help, it is possible to easily detect the presence of SBS in modified bitumen. However, quantitative analysis is an issue that requires research. Currently, there are no standard guidelines.

Studies indicate that SBS levels, up to approximately 6%, promote the development of a critical elastic network, which imparts maximum strength and rheological stability to the binder. Exceeding this threshold leads to phase separation between the polymer and the bituminous matrix.

A range of analytical techniques has been employed to determine polymer content in PMBs. Rheological methods such as dynamic shear rheometer (DSR), multiple stress creep and recovery (MSCR), and viscosity measurements can indirectly reflect polymer concentration, but they do not provide a direct quantitative assessment [13,14]. Moreover, the relationship is not always linear, as the response depends strongly on the base bitumen.

Dissolution and phase-separation methods rely on selectively dissolving the bituminous fraction to isolate the solid polymer phase. They are most suitable for polymers such as APP. However, their broader application is limited by significant drawbacks, including partial dissolution of SBS, polymer particle aggregation, lengthy analysis times, and the requirement for toxic solvents.

Gel permeation chromatography (GPC) enables the separation of components according to their molecular weight. The technique provides high analytical accuracy but requires expensive instrumentation and a high level of operator expertise.

Recent studies indicate that infrared spectroscopy is the most efficient and accessible method for quantitatively determining polymer content in PMBs [14].

The present article is based on scientific findings reported in recent research.

The aim of this study is to develop and experimentally validate a method for quantifying the SBS polymer modifier in polymer-modified bituminous binders using FTIR-ATR spectroscopy, with the construction of a calibration curve suitable for routine laboratory quality control.

Research objectives:

1. Prepare a series of calibration PMB samples with SBS contents ranging from 1% to 4% and determine their physical and mechanical properties in accordance with national standards (ST RK) [15,16,17,18,19,20,21,22,23,24,25,26,27].
2. Acquire IR spectra of both calibration and test samples and identify characteristic polymer absorption bands (966; 699–760; 1600 cm^−1^) along with reference bitumen bands.
3. Construct a first-order calibration model linking the intensity of analytical peaks to the actual polymer content.
4. Determine the true polymer concentration in the examined PMB samples and evaluate the accuracy and reproducibility of the proposed method.
5. Establish the relationship between actual polymer content and the physical–mechanical performance of the resulting PMBs.

Infrared spectroscopy offers several clear advantages: high reproducibility, suitability for quantitative analysis, no requirement for selective solvents, minimal sample preparation, and the ability to distinguish between different polymer types. As noted in the literature, the technique enables the identification of SBS, SBR, and EVA by isolating their characteristic absorption bands [9].

For SBS, the following absorption features are considered diagnostic:

- 966 cm^−1^—C=C deformation of the butadiene units;
- 699–760 cm^−1^—aromatic ring vibrations of styrene;
- 1600 cm^−1^—aromatic rings of the styrene blocks;
- 1460 cm^−1^—methylene groups of bitumen (used as a reference line).

Prior studies have demonstrated that the intensity of SBS-related absorption bands correlates linearly with polymer concentration, which makes the method suitable for constructing calibration models.

## 2. Materials and Methods

The infrared-based method for determining polymer content described in PNST 960–2023 [27] represents a specialized regulatory standard that, for the first time in Russian practice, formalizes a direct instrumental procedure for quantifying the mass fraction of polymer modifiers in bituminous binders. PNST 860–2023 [28] specifies requirements for the equipment, measurement conditions, sample preparation, selection of analytical bands, calibration procedures, and calculation of results. The standard also defines criteria for repeatability, reproducibility, and acceptable differences between parallel measurements. The method is categorized as a rapid, non-destructive analytical technique that does not require labor-intensive chemical extraction or complex sample preparation, making it particularly well suited for production-level quality control.

**Figure 1:**
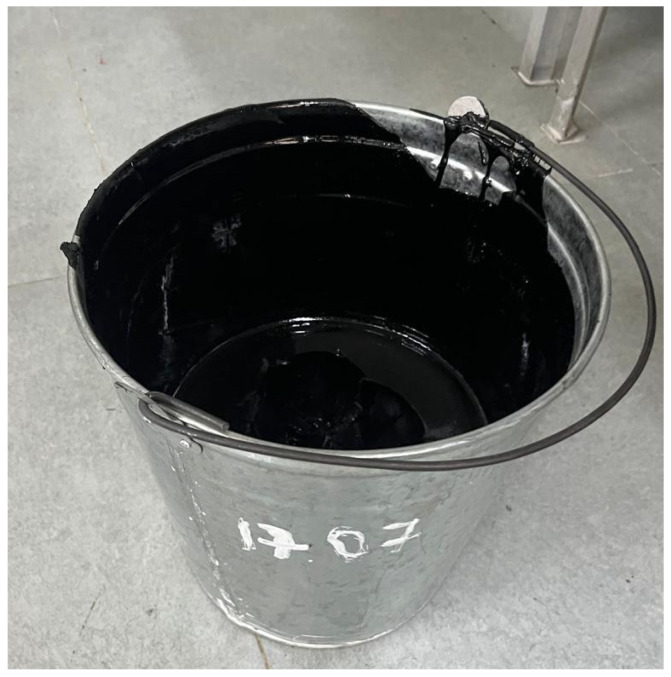
Road bitumen of grade 100/130.

For the experimental work, base road bitumen of grade 100/130 (PNHZ, Pavlodar, Kazakhstan) (Figure 1) and a polymer modifier used in the production of polymer-modified binders for asphalt mixture manufacturing were employed as initial materials. The selection of bitumen grade and polymer type was guided by their prevalence in road construction practice as well as their compliance with applicable regulatory standards [29,30,31,32].

The physical and mechanical properties of both unmodified bitumen and polymer-modified binders were evaluated according to the requirements of ST RK 1373 [15] and ST RK 2534 [16]. The analysis was performed using instrumentation appropriate for determining the respective performance characteristics.

**Figure 2:**
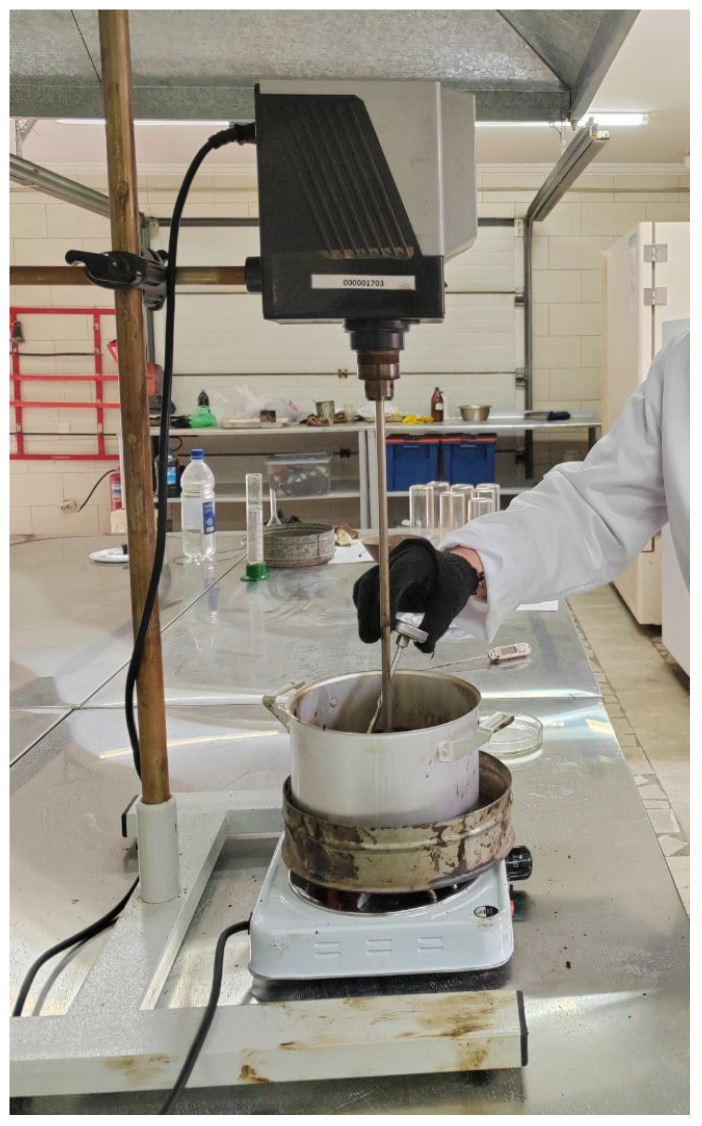
The process of preparation of polymer-modified binder.

For the preparation of polymer-modified binders, the bitumen was first brought to a fluid state and dehydrated by holding it in a drying oven at a temperature of (105 ± 5) °C. After dehydration, the bitumen was heated to 150–160 °C, and the polymer was gradually introduced in amounts ranging from 1% to 4% while continuously mixing with a laboratory stirrer. Mixing was maintained for two hours at 165–170 °C to achieve partial dissolution of the polymer in Figure 2.

**Figure 3:**
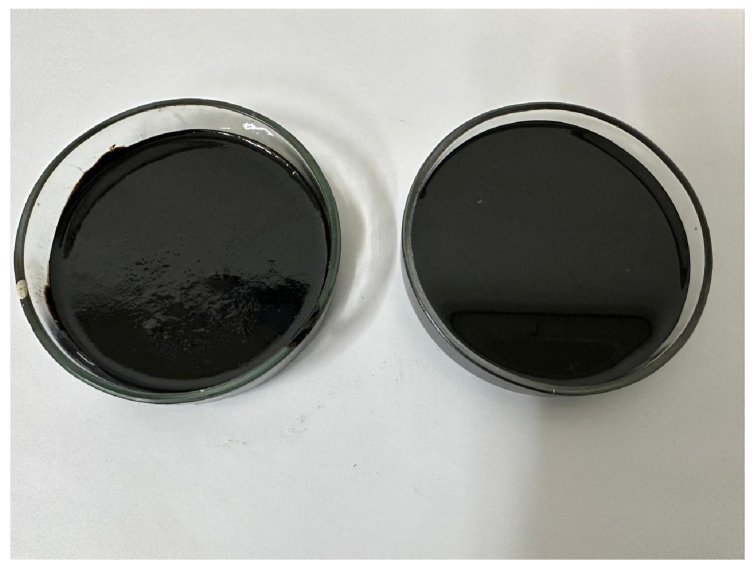
Bitumen with SBS polymer and initial bitumen (according to the figure).

To ensure a more complete dissolution, the mixture was additionally homogenized for 10 min using a mechanical homogenizer, after which it was mixed for another hour at the same temperature, resulting in a homogeneous and completely dissolved PMB and for comparison with the initial bitumen shown in Figure 3.

**Figure 4:**
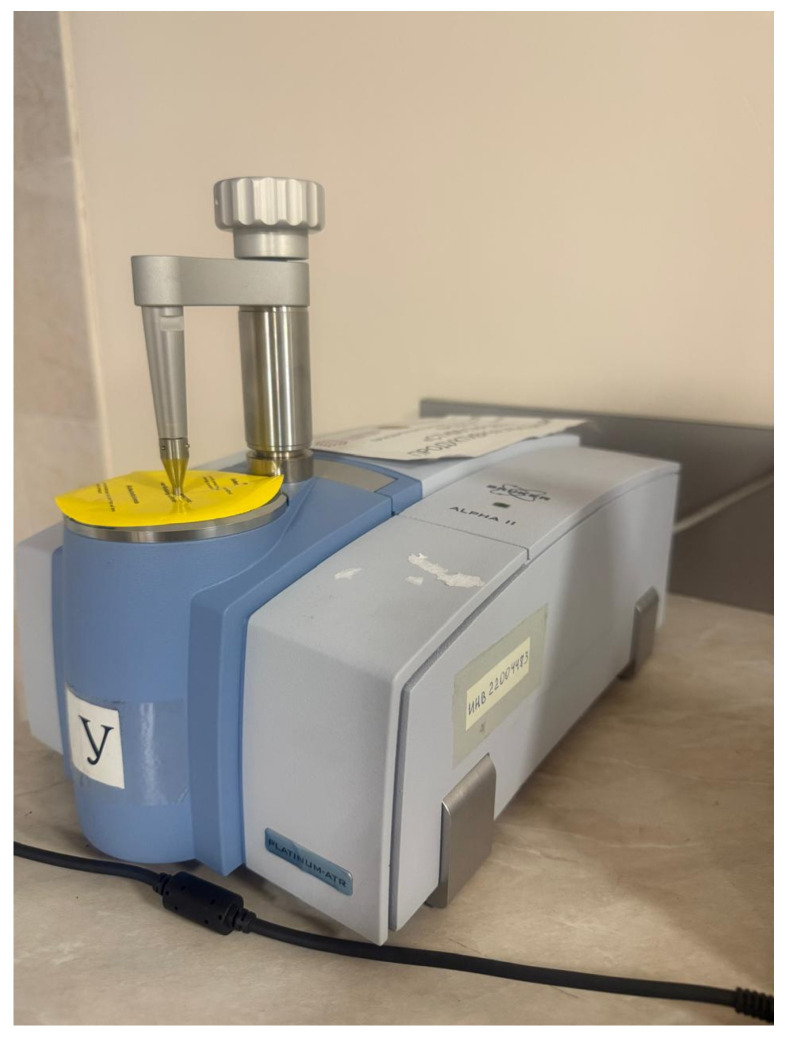
FTIR Spectrometer ALPHA II (Bruker, Karlsruhe, Germany).

The prepared samples were then used for spectroscopic analysis. The analytical procedure involves constructing a first-order calibration relationship obtained by comparing the absorption features in the IR spectra with the known SBS (Sibur, Moscow, Russia) content (1–4%) used in preparing the calibration samples. This calibration curve is subsequently applied to determine the actual mass fraction of polymer in the tested PMB specimens. The quantitative determination was performed using Fourier-transform infrared spectroscopy (FTIR) with an ALPHA II spectrometer manufactured by Bruker (Karlsruhe, Germany) (Figure 4).

For instrument calibration, PMB samples with predefined SBS contents ranging from 1% to 4% were prepared to establish the concentration interval required for constructing a first-order calibration curve.

The main stages of the test procedure were as follows:

- Recording the spectra of calibration samples and documenting the intensities of characteristic polymer peaks along with the reference peaks of bitumen;
- Constructing a first-order calibration relationship between the ratio of peak areas (or heights) and the known polymer content;
- Acquiring spectra of the test samples and determining the actual polymer mass fraction using the calibration curve.

The integration ranges for the analytical peaks were selected in accordance with the standard recommendations of PNST 860–2023 [28] and relevant international methodologies. For SBS, the diagnostic peaks used in the analysis are located near 966 cm^−1^ and 699–700 cm^−1^.

## 3. Results

Table 1 shows the results of tests on the physical and mechanical properties of bituminous binder grade BND 100/130.

Table 2 shows the results of tests on the physical and mechanical properties of bituminous binder modified with SBS polymer added in quantities of 1%, 2%, 3% and 4% of the bitumen mass.

**Figure 5:**
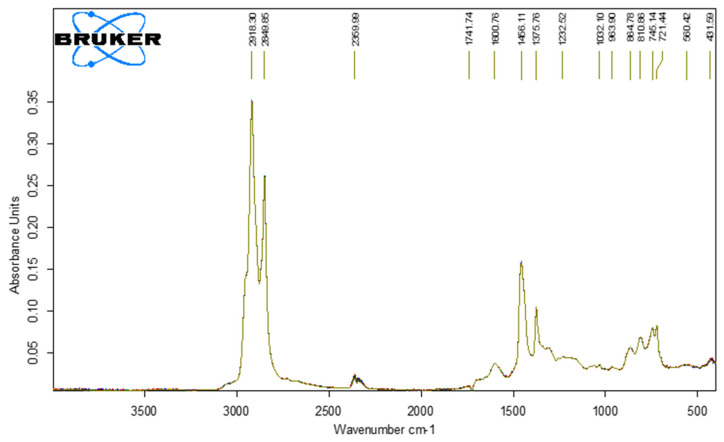
IR spectrum of the original bituminous binder (PNHZ).

Each bitumen sample was brought to a fluid state prior to analysis and dehydrated in a drying oven at a temperature of (105 ± 5) °C to eliminate the influence of moisture on the spectroscopic results. Figure 5 shows the spectrum of unmodified bitumen produced by Pavlodar Oil Refinery and Chemical Plant LLP (PNHZ).

**Figure 6:**
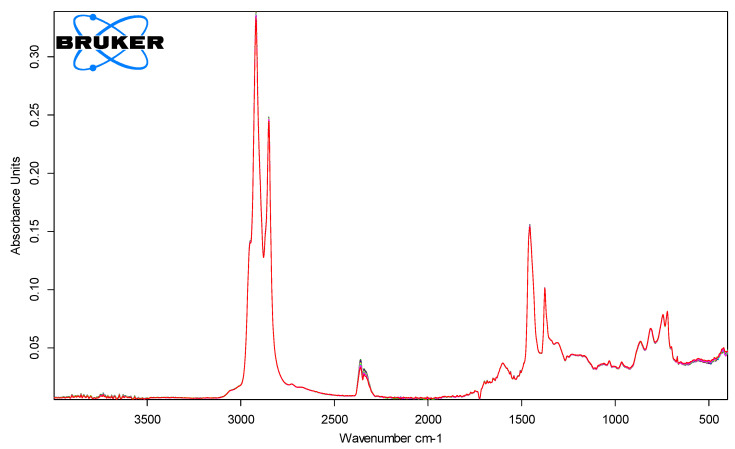
IR spectra of PMB with 1% SBS polymer content (10 parallel measurements).

**Figure 7:**
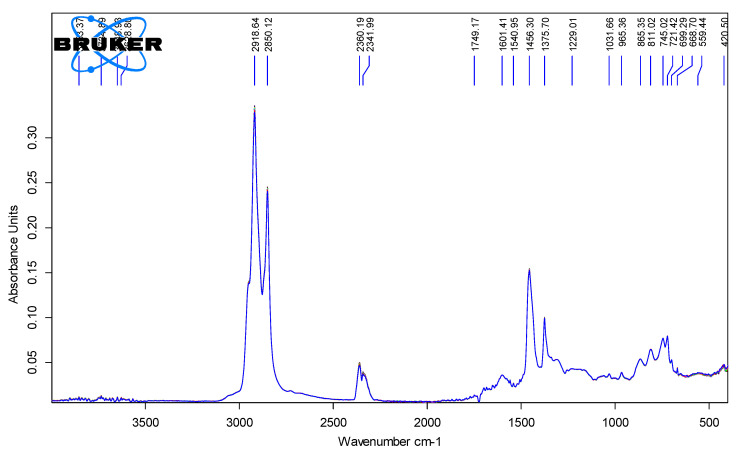
IR spectra of PMB with 2% polymer content (10 parallel measurements).

**Figure 8:**
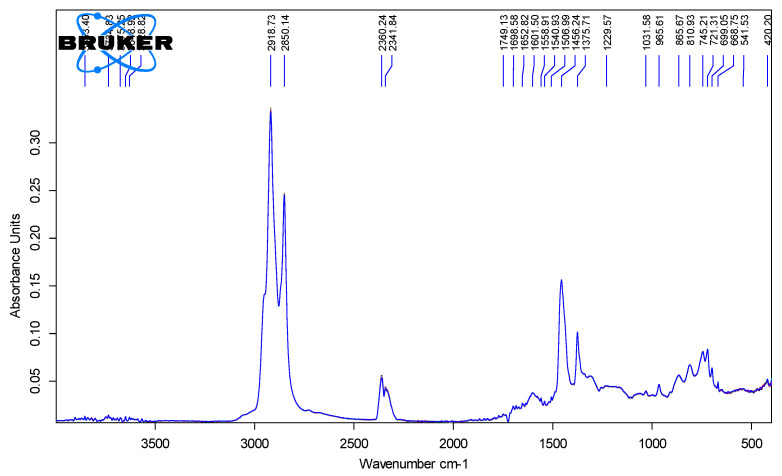
IR spectra of PMB containing 3% polymer (10 parallel measurements).

**Figure 9:**
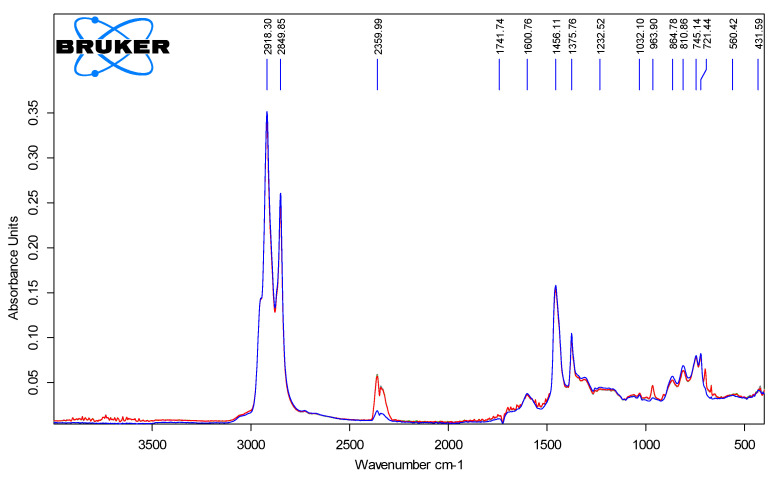
IR spectra of PMB containing 4% polymer (10 parallel measurements).

Figure 6, Figure 7, Figure 8 and Figure 9 show the spectra of PMB with 1–4% SBS polymer content.

**Figure 10:**
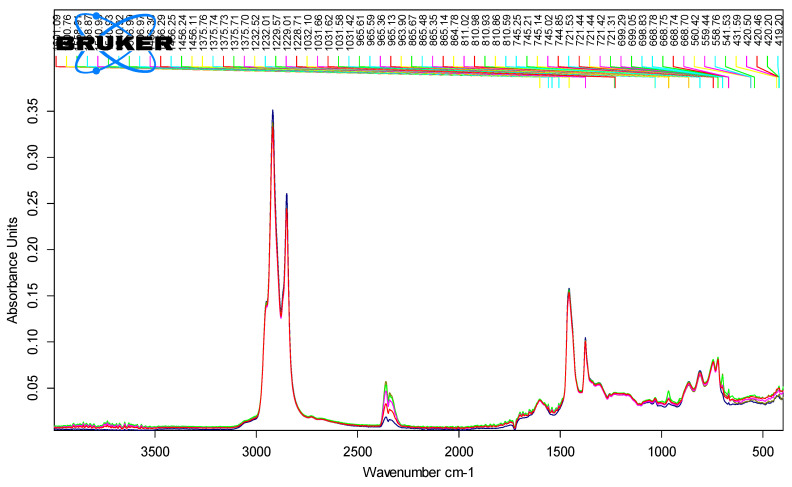
IR spectra of PMB samples with polymer content ranging from 1% to 4%.

Figure 10 shows the spectra of all modified polymer bitumen binders with an SBS polymer content of 1–4%.

## 4. Discussion

The results obtained during the tests demonstrate that the physical and mechanical properties of the base bituminous binder comply with the requirements of the ST RK 1373 standard [15]. Based on this binder, PMB samples were prepared for subsequent investigation of their structural characteristics.

Analysis of the data presented in Table 2 shows that modification of the bituminous binder with SBS polymer at concentrations of 3–4% leads to a pronounced increase in softening point, penetration depth, and elasticity. These changes enhance the material’s resistance to elevated temperatures and dynamic loading.

Examination of the FTIR spectra of SBS-modified PMBs revealed three distinct absorption peaks associated with the presence of SBS polymer structures. The first peak corresponds to styrene vibrations at approximately 700 cm^−1^, the second to butadiene groups near 970 cm^−1^, and the third to another styrene-related band around 1380 cm^−1^.

For each of these characteristic peaks, a separate baseline was constructed by connecting two spectral points near the corresponding wavenumber. This allowed accurate determination of the area under the peak and, consequently, the absorption intensity proportional to the polymer content in the sample.

**Figure 11:**
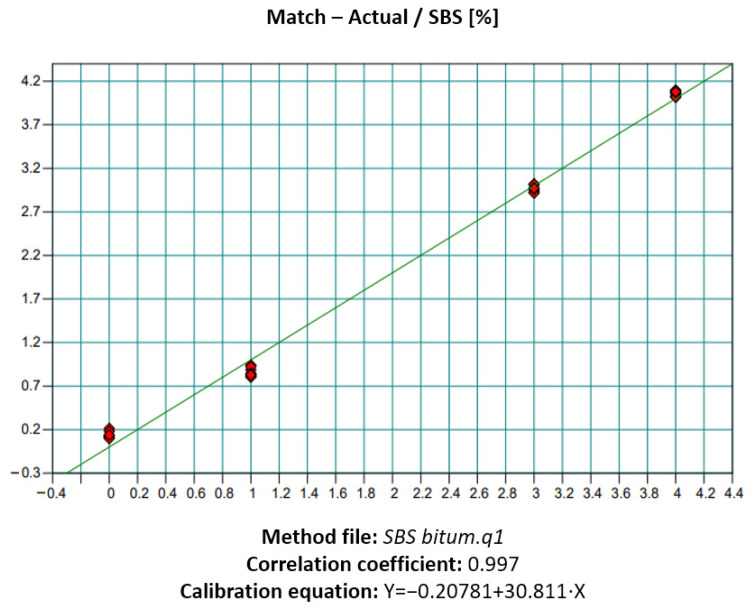
Calibration curve of PMB (SBS).

To quantify the polymer concentration in PMBs, a calibration curve was developed (Figure 11). This curve relates the optical density (absorption intensity) of the characteristic peaks to the known polymer content in the reference samples.

Figure 11 presents the calibration relationship between the intensity of the characteristic IR peak and the SBS concentration in the PMB. The resulting linear fit is described by the following equation:

*Y* = −0.20781 + 30.811 ∙ *X* (1)

where Y is the polymer content (%); –0.20781 and 30.811 are the coefficients of the linear regression equation; and X is the peak intensity.

## 5. Conclusions

In the course of this research project, a comprehensive investigation of polymer-modified bituminous binders was carried out with the objective of quantitatively determining the polymer modifier content and evaluating its influence on the physical and mechanical properties of bitumen. The analysis of applicable regulatory documents, including PNST 960–2023, demonstrated that this standard is modern, well-adapted to local conditions, and provides a direct, accurate, and reproducible method for determining the mass fraction of polymer in bituminous binders. This makes it highly suitable for use in laboratories across Kazakhstan, both in research settings and in production control.

The experimental work involved the preparation of calibration and test samples of polymer-modified bitumen, FTIR-ATR spectroscopic measurements, construction of calibration curves, and calculation of the actual polymer content. The results showed high accuracy and reproducibility of the method and revealed a clear correlation between polymer concentration and improvements in the performance characteristics of the bituminous binder, including increases in softening point, penetration depth, and elasticity.

Statistical evaluation of the experimental data confirmed the reliability of the findings, identified consistent trends in the variation in physical and mechanical properties with increasing polymer content, and enabled the justification of optimal concentration ranges for polymer modifiers to achieve the required performance characteristics of asphalt mixtures.

The study covered a specific range of 1 to 4% SBS polymer content, so conclusions regarding the properties of bitumen at higher or lower concentrations require further investigation. The work provides practical information for the quality control of PMB and for optimizing the composition of asphalt mixtures, while future research could focus on expanding the range of polymers, concentrations, and operational conditions.

## Figures and Tables

**Table 1:** Physical and mechanical properties of the base bitumen grade BND 100/130.

No.	Property	Unit	Test Standard	Requirement	Actual Value
1	Penetration at: 25 °C; 0 °C (not less than)	×0.1 mm	ST RK 1226-2003 [17]	101–130; 30	115; 39
2	Softening point (ring and ball), min	°C	ST RK 1227-2003 [18]	43	47
3	Ductility at: 25 °C; 0 °C, min	cm	ST RK 1374-2005 [19]	70; 4.0	145; 5.2
4	Dynamic viscosity at 60 °C, min	Pa·s	ST RK 1211-2003 [20]	120	138
5	Kinematic viscosity at 135 °C, min	mm^2^/s	ST RK 1210-2003 [21]	180	315
6	Flash point, min	°C	ST RK 1804-2008 [22]	230	290
7	Fraass breaking point, max	°C	ST RK 1229-2003 [23]	–22	–27
8	Penetration index	—	ST RK 1373-2013 [15]	–1.0 to +1.0	–0.02
9	Solubility, min	%	ST RK 1228-2003 [24]	99.0	99.9
10	Paraffin content, max	%	ST RK 1230-2003 [25]	2.5	0.98
11	Aging resistance after heating at 163 °C:				
11.1	– Mass change, max	%	ST RK 1224-2003 [26]	0.7	0.02
11.2	– Penetration at 25 °C after aging, min (% of original)	%	ST RK 1552-2006 [27]	50	75
11.3	– Ductility at 25 °C after aging, min	cm	ST RK 1374-2005 [19]	80	94
11.4	– Softening point change, max	°C	ST RK 1227-2003 [18]	7.0	5.0
11.5	– Increase coefficient of dynamic viscosity at 60 °C, max	—	p. 8.2.4 ST RK 1373-2013 [15]	2.5	2.4

**Table 2:** Physical and mechanical properties of PMBs containing 1–4% SBS (SIBUR production).

No.	Property (Unit)	Standard Requirement for Grades		SBS Content, %			
		35/50	50/70	1.0	2.0	3.0	4.0
1	Penetration at 25 °C, ×0.1 mm	35–50	51–70	64	52	49	39
2	Softening point (ring and ball), °C, min	65	62	54	60	69	77
3	Ductility at 25 °C, cm, min	15	20	107	78	72	43
4	Fraass breaking point, °C, max	−15	−16	−20	−21	−21	−22
5	Elasticity at 25 °C, %, min	60	60	55	74	86	95
Aging resistance after heating at 163 °C (ST RK 1552)							
6	Mass change, %, max	0.5	0.5	0.16	0.17	0.17	0.18
7	Softening point change (ring and ball), °C, max: increase/decrease	5/6	—	3.0/4.0	4.0/6.0	—	—
8	Ductility at 25 °C after aging, cm	Not regulated	—	86	44	40	33
9	Elasticity at 25 °C after aging, %, min	50	50	70	78	80	83

## Data Availability

The raw data supporting the conclusions of this article will be made available by the authors on request.

## Abbreviations

The following abbreviations are used in this manuscript:

PMB	Polymer Modified Bitumen
FTIR-ATR	Fourier Transform Infrared Spectroscopy using Attenuated Total Reflectance Mode
SBS	Styrene-Butadiene-Styrene
SBR	Styrene-Butadiene Rubber
APP	Amorphous Polypropylene Polymers
DSR	Dynamic Shear Rheometer
MSCR	Multiple Stress Creep and Recovery
IR	Infrared
PNHZ	Pavlodar Oil Refinery and Chemical Plant LLP
BND	Bituminous Binder Grade

## References

[1] Alibayeva A., Amirbayev Y., Mukhambetkaliyev K., Alizhanov D., Zhumamuratov M., Lukpanov R., Zhumagulova A., Dyussembinov D., Smagulova M. (2025). Evaluation of temperature parameters of lowgrade modified bitumen in comparison with high grade bitumen. GEOMATE J..

[2] Shakhmov Z., Zhumagulova A., Kosparmakova S., Kozhahmet A., Kabdrashit J. (2023). Properties of modified bitumen in road construction. Technobius.

[3] Teltayev B., Radovskiy B., Seilkhanov T., Oliviero Rossi C., Amirbayev E. (2022). Low and high temperature characteristics of compounded and modified bitumens. Colloids Surf. Physicochem. Eng. Asp..

[4] Zhurinov M.Z., Teltayev B.B., Amirbayev Y.D., Begaliyeva S.T., Alizhanov D.A. (2021). Mechanical characteristics of road compounded bitumen at lowtemperatures. News Natl. Acad. Sci. Repub. Kazakhstan Ser. Geol. Tech. Sci..

[5] Zofka A., Maliszewska D., Maliszewski M., Boratyński J. (2015). Application of FTIR ATR method to examine the polymer content in the modified bitumen and to assess susceptibility of bitumen to ageing. Roads Bridges Drogi i Mosty.

[6] Teltayev B., Amirbayev E., Radovskiy B. (2022). Evaluating the Effect of Polymer Modification on the Low-Temperature Rheological Properties of Asphalt Binder. Polymers.

[7] Soenen H., Vansteenkiste S., Kara De Maeijer P. (2020). Fundamental Approaches to Predict Moisture Damage in Asphalt Mixtures: State-of-the-Art Review. Infrastructures.

[8] Deller Z., Maniam S., Giustozzi F. (2022). Sample Preparation and Analytical Methods for Identifying Organic Compounds in Bituminous Emissions. Molecules.

[9] Chen J.-S., Liao M.-C., Lin C.-H. (2003). Determination of polymer content in modified bitumen. Mater. Struct..

[10] Sun D.Q., Zhang L.W. (2013). A Quantitative Determination of Polymer Content in SBS Modified Asphalt. Part I: State of the Art. Pet. Sci. Technol..

[11] Emtiaz M., Imtiyaz M.N., Majumder M., Idris I.I., Mazumder R., Rahaman M.M. (2023). A Comprehensive Literature Review on Polymer-Modified Asphalt Binder. CivilEng.

[12] Ratajczak M., Wilmański A. (2020). Evaluation of laboratory methods of determination of SBS content in polymer-modified bitumens. Materials.

[13] Sengoz B., Isikyakar G. (2008). Analysis of styrene-butadiene-styrene polymer modified bitumen using fluorescent microscopy and conventional test methods. J. Hazard. Mater..

[14] Rødland E.S., Samanipour S., Rauert C., Okoffo E.D., Reid M.J., Heier L.S., Lind O.C., Thomas K.V., Meland S. (2022). A novel method for the quantification of tire and polymer-modified bitumen particles in environmental samples by pyrolysis gas chromatography mass spectroscopy. J. Hazard. Mater..

[15] (2013). Bitumen and Bitumen Binders. Petroleum Road Bitumen Is Viscous. Technical Conditions. (amendments 1,2).

[16] (2014). Bitumen and Bitumen Binders. Modified Petroleum Bitumen, Road Bitumen. Technical Conditions.

[17] (2003). Bitumen and Bitumen Binders. The Method of Determining the Depth of Needle Penetration.

[18] (2003). Bitumen and Bitumen Binders. Determination of the Softening Point by the Ring and Ball Method.

[19] (2005). Bitumen and Bitumen Binders. The Method of Determining Extensibility.

[20] (2003). Bitumen and Bitumen Binders. Dynamic Viscosity Determination Method.

[21] (2003). Bitumen and Bitumen Binders. Method for Determining Kinematic Viscosity. [Electronic Resource].

[22] (2008). Bitumen and Bitumen Binders. Methods for Determining Flash and Ignition Temperatures in an Open Crucible.

[23] (2003). Petroleum Bitumen and Bitumen Binders. Fraas Brittleness Temperature Determination Method.

[24] Bitumen and Bitumen Binders. Solubility Determination Method. (DIN EN 12592:2000 MOD) [Electronic Resource]. PARAGRAPH Information System.

[25] (2003). Petroleum Bitumen. Methods for Determining the Content of Paraffin.

[26] (2003). Bitumen and Bitumen Binders. Methods for Determining Resistance to Aging Under the Influence of Heating and the Air Environment.

[27] (2006). Bitumen and Bitumen Binders. Methods for Determining Weight Changes After Warming up.

[28] (2023). Public Roads. Petroleum Bitumen Binders. A Method for Determining the Amount of Polymer Using the Infrared Spectrum; (Approved and Put into Effect by Order of the Federal Agency for Technical Regulation and Metrology Dated 09/27/2023 N 36-pnst).

[29] (2023). Standard Specification for Performance-Graded Asphalt Binder.

[30] (2010). Bitumen and Bituminous Binders—Specification Framework for Polymer Modified Bitumens.

[31] (2022). Standard Method of Test for Polymer Content of Polymer-Modified Emulsified Asphalt Residue and Asphalt Binders.

[32] (2023). Standard Specification for Performance-Graded Asphalt Binder Using Multiple Stress Creep Recovery (MSCR) Test.

